# Endotoxin Activity in Patients With Extracorporeal Membrane Oxygenation Life Support: An Observational Pilot Study

**DOI:** 10.3389/fmed.2021.772413

**Published:** 2021-11-29

**Authors:** Chen-Tse Lee, Chih-Hsien Wang, Wing-Sum Chan, Yun-Yi Tsai, Tzu-Jung Wei, Chien-Heng Lai, Ming-Jiuh Wang, Yih-Sharng Chen, Yu-Chang Yeh

**Affiliations:** ^1^Department of Anesthesiology, National Taiwan University Hospital, College of Medicine, National Taiwan University, Taipei, Taiwan; ^2^Department of Surgery, National Taiwan University Hospital, College of Medicine, National Taiwan University, Taipei, Taiwan; ^3^Department of Anesthesiology, Far Eastern Memorial Hospital, New Taipei, Taiwan

**Keywords:** critical care, endotoxin, extracorporeal membrane oxygenation (ECMO), survival, infection

## Abstract

**Background:** Extracorporeal membrane oxygenation (ECMO) life support has become an integral part of intensive care. The endotoxin activity assay (EAA) is a useful test to measure endotoxemia severity in whole blood. To date, no information is available regarding the EAA levels and their effect on clinical outcomes in critically ill patients with ECMO support.

**Methods:** This prospective observational pilot study enrolled adult critically ill patients with ECMO support from August 2019 to December 2020. The EAA levels were measured within 24 h (T1), and at 25–48 (T2), 49–72 (T3), and 73–96 h (T4) after ECMO initiation. This study primarily aimed to investigate the incidence of high EAA levels (≥0.6) at each time point. Subsequent exploratory analyses were conducted to compare the EAA levels of venoarterial ECMO (VA-ECMO) patients between 30-day survivors and non-survivors. *Post*-*hoc* analysis was performed to compare the clinical outcomes of VA-ECMO patients with elevated EAA levels at T3 (vs. T1) and those without elevated EAA levels.

**Results:** A total of 39 VA-ECMO patients and 15 venovenous ECMO (VV-ECMO) patients were enrolled. At T1, the incidence of high EAA level (≥0.6) was 42% in VV-ECMO patients and 9% in VA-ECMO patients (*P* = 0.02). At T2, the incidence of high EAA level was 40% in VV-ECMO patients and 5% in VA-ECMO patients (*P* = 0.005). In VA-ECMO patients, EAA levels at T3 were significantly higher in 30-day non-survivors than in survivors (median [interquartile range]: 0.49 [0.37–0.93] vs. 0.31 [0.19–0.51], median difference 0.16 [95% confidence interval [CI], 0.02–0.31]; *P* = 0.024). Moreover, VA-ECMO patients with elevated EAA levels at T3 (vs. T1) had lower 30-day survival than patients without elevated EAA levels (39 vs. 83%, *P* = 0.026) and fewer ECMO free days by day 30 (median: 3 vs. 23 days, median difference 12 days [95% CI, 0–22]; *P* = 0.028).

**Conclusions:** A certain proportion of patients experienced high EAA levels (≥0.6) after VV-ECMO or VA-ECMO initiation. VA-ECMO patients with an elevated EAA level at 49–72 h were associated with poor clinical outcomes.

## Introduction

Extracorporeal membrane oxygenation (ECMO) life support has become an integral part of intensive care (1), but contingent infection increases morbidity and mortality in patients receiving ECMO (2, 3). Venovenous ECMO (VV-ECMO) is primarily indicated for patients with acute respiratory distress syndrome (ARDS), which is frequently associated with pneumonia and sepsis. Venoarterial ECMO (VA-ECMO) is primarily used for supporting patients with refractory cardiogenic shock. According to our clinical experience, fever and sepsis after VA-ECMO are common. In addition to some common nosocomial infections, severe hypoperfusion can disrupt the intestinal barrier and result in bacteremia and endotoxemia (4, 5).

High endotoxin levels are associated with poor survival outcomes in critically ill patients (6). A novel immunoassay using neutrophil-dependent chemiluminescence, the endotoxin activity assay (EAA), was developed to measure endotoxin activity in whole blood (7). Previous research demonstrated that an EAA level ≥0.6 was strongly associated with the development of septic shock and mortality in critically ill patients (8). However, the incidence and effects of high endotoxin levels on clinical outcomes in critically ill patients on ECMO support remain unclear. The primary goal of this pilot study was to investigate that how many critically ill patients with VV-ECMO or VA-ECMO support would have an EAA level higher than 0.6. In addition, for these patients with VV-ECMO or VA-ECMO support, the secondary goals of this study were to investigate whether survivors and non-survivors were different in EAA levels, and to investigate whether patients with or without elevated EAA levels had different clinical outcomes within 30 days after ECMO initiation.

## Materials and Methods

### Patients

This prospective observational pilot study was approved by the Research Ethics Committee of National Taiwan University Hospital (approval number 201811061RINC), and was registered at clinicaltrials.gov (NCT03978728). This study was conducted between August 2019 and December 2020 and was reported in accordance with the Strengthening the Reporting of Observational Studies in Epidemiology (STROBE) guidelines (9). Adult critically ill patients who underwent VV-ECMO or VA-ECMO were evaluated for eligibility within 24 h of ECMO initiation. Patients were excluded if they aged younger than 20 years or elder than 90 years, had do-not-resuscitate orders, underwent extracorporeal endotoxin adsorption treatment during the study period, had no blood sample obtained within 48 h of ECMO initiation, or did not speak Taiwanese natively. Informed consent was obtained from the legally authorized representatives of all participants before enrollment. A total of 196 critically ill patients receiving ECMO support were assessed for the eligibility of this study.

### ECMO Components

The implementation and principal components of ECMO for all enrolled patients were as described in our previous study (10). The VA-ECMO was placed in the femoral vein (21–23 French) and artery (15–19 French), and VV-ECMO was achieved using femoral inflow (21–23 French) and jugular outflow (15–19 French). To avoid malperfusion of the distal limb, an antegrade distal perfusion catheter was used when the mean pressure of the superficial femoral artery was <50 mmHg (11). All enrolled patients received standard ECMO management and intensive care unit (ICU) care. Heparin was continuously administered to maintain an activated clotting time of 160–180 s if no active bleeding or other complications were observed. Antibiotic prophylaxis with vancomycin and ceftazidime were administered shortly after ECMO to patients without antibiotic use; the antibiotics were subsequently downgraded in accordance with institutional protocols.

### Data Collection

Demographic data including age, sex, height, body weight, indications for ECMO support, Survival after VA-ECMO (SAVE) score, Respiratory ECMO Survival Prediction (RESP) score, and Acute Physiology and Chronic Health Evaluation (APACHE) II score were recorded after enrollment. Blood samples were obtained at four time points after ECMO initiation: within 24 h (T1) and at 25–48 (T2), 49–72 (T3), and 73–96 h (T4). White blood cell (WBC) counts; lactate, procalcitonin, diamine oxidase (DAO), and cystatin C levels; and EAA levels were examined. The inotropic score (IE) was calculated at the four time points as 100 × epinephrine dose (μg/kg/min) + 100 × norepinephrine dose (μg/kg/min) + dopamine dose (μg/kg/min) + dobutamine dose (μg/kg/min) (12). The duration of ECMO support and survival status at 30 days were recorded.

### Measurements of EAA Levels and Blood Inflammatory Biomarkers

The blood samples were collected from the peripheral arterial line. An EAA measurement was performed within 3 h after blood sample collection using the EAA Kit (Spectral Medic al Inc., Toronto, Canada) according to the manufacturer's instructions. An EAA level ≥0.6 was considered high. Biomarkers were measured using commercially available enzyme-linked immunosorbent assay (ELISA) kits according to the manufacturer's instructions. Procalcitonin was measured using the Human Procalcitonin SimpleStep ELISA Kit (Abcam, Cambridge, UK). DAO was measured using the IDK DAO ELISA Kit (Immundiagnostik AG, Bensheim, Germany). Finally, cystatin C was measured using the Cystatin C (CST3) (Human) ELISA Kit (Biovision, Inc., CA, USA).

The primary goal was to obtain the EAA levels, particularly the incidence of high EAA levels (≥0.6), at each time point in patients with VV-ECMO or VA-ECMO. Exploratory analyses were conducted to compare EAA levels between 30-day survivors and non-survivors. *Post-hoc* exploratory analysis was performed to compare the clinical outcomes between VA-ECMO patients with and without an elevated EAA level at T3 (vs. T1).

### Statistical Analysis

Data were analyzed in SPSS Version 27.0 (IBM, Armonk, NY, USA). Continuous variables are reported as medians (interquartile ranges) and were compared using the Mann–Whitney *U* test; median differences (95% confidence intervals [CIs]) between groups were calculated using the Hodges–Lehmann estimator. Categorical variables are reported as numbers (percentages) and were compared using chi-square tests or Fisher's exact tests as appropriate. The Kaplan–Meier survival curve was employed to visualize the survival differences between groups, which were tested using log-rank tests. A *P* < 0.05 indicated a significant difference.

## Results

### Patient Characteristics and EAA Levels

**Figure 1:**
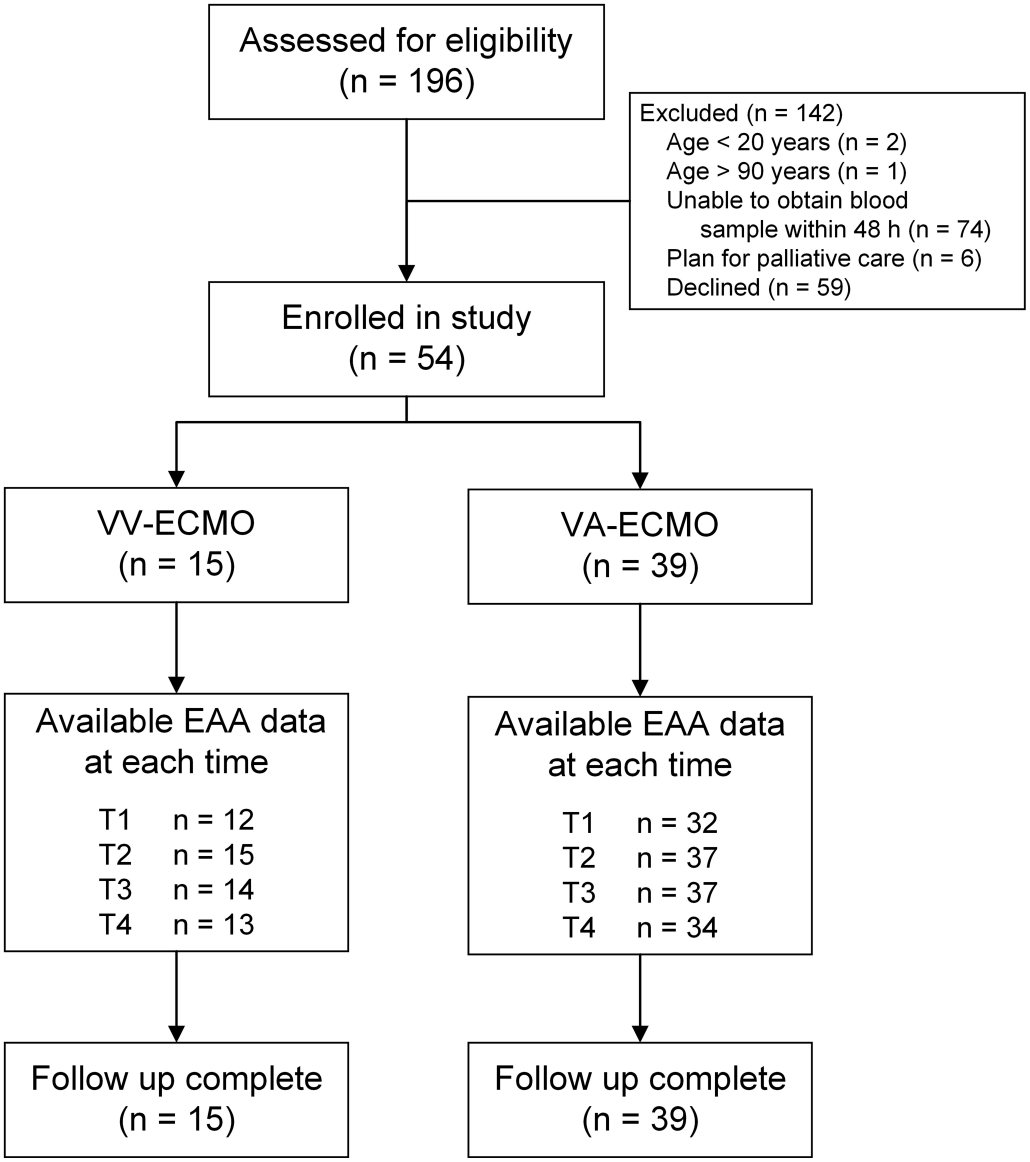
Flow diagram. EAA, endotoxin activity assay; VA-ECMO, venoarterial extracorporeal membrane oxygenation; VV-ECMO, venovenous extracorporeal membrane oxygenation.

**Figure 2:**
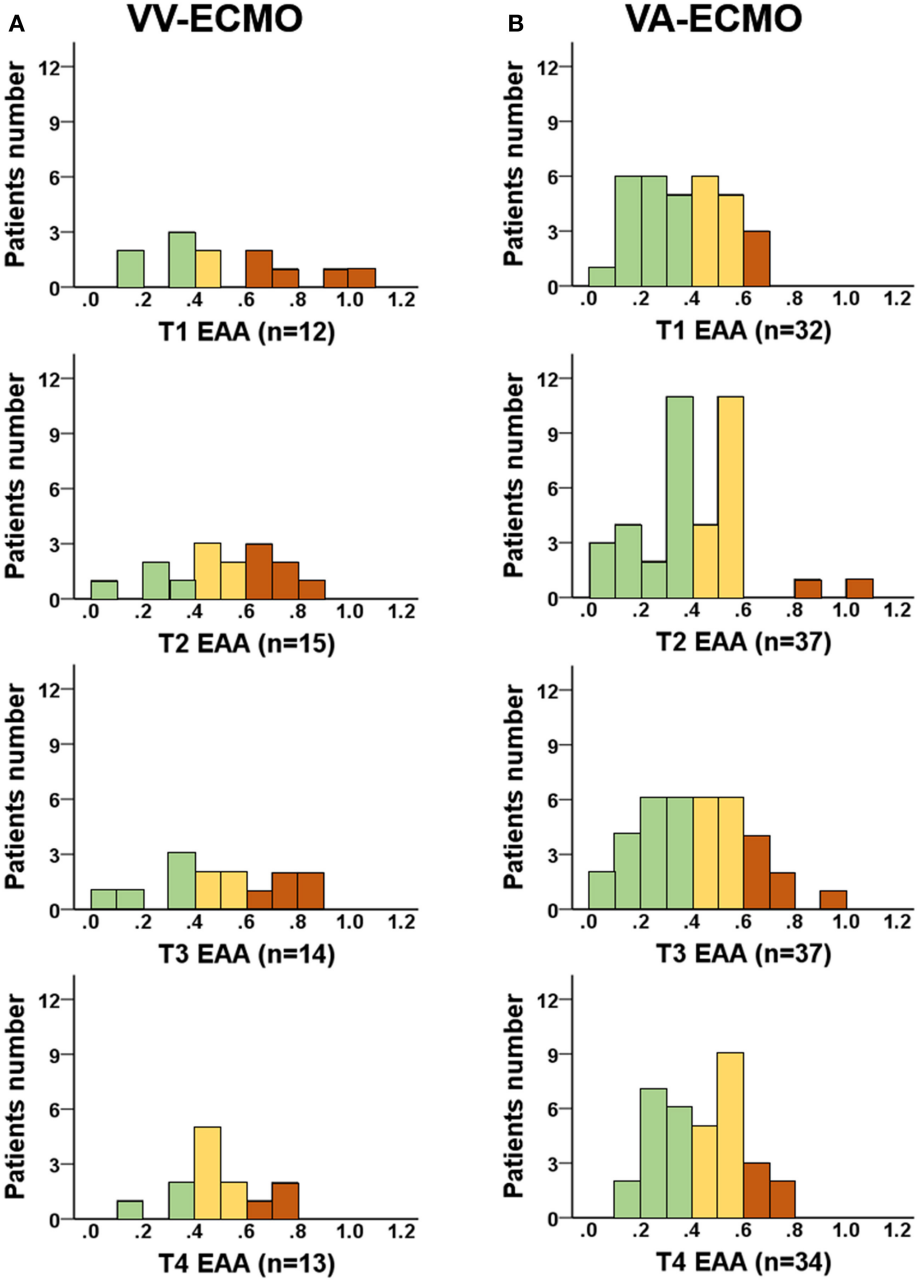
EAA levels in VA-ECMO or VV-ECMO patients. EAA levels were measured at four time points: within 24 h (T1) and 25–48 (T2), 49–72 (T3), and 73–96 h (T4) after ECMO initiation. **(A)** EAA levels in VV-ECMO patients; **(B)** EAA levels in VA-ECMO patients. Brown column stands for high EAA level (≥0.6); yellow column stands for intermediate EAA level (0.4 to 0.6); green column stands for low EAA level (<0.4). EAA, endotoxin activity assay; VA-ECMO, venoarterial extracorporeal membrane oxygenation; VV-ECMO, venovenous extracorporeal membrane oxygenation.

After eligibility assessment and informed consent, 39 VA-ECMO patients and 15 VV-ECMO patients were enrolled (Figure 1). The patient characteristics, indications for ECMO, and EAA levels are summarized in Table 1. The 30-day survival rates were 54 and 73% in VA-ECMO and VV-ECMO patients, respectively. The EAA levels in VV-ECMO patients and VA-ECMO patients are presented in Figure 2. At T1, the incidence of high EAA levels (≥0.6) was 42% in VV-ECMO patients and 9% in VA-ECMO patients (*P* = 0.02). At T2, the incidence of high EAA levels was 40% in VV-ECMO patients and 5% in VA-ECMO patients (*P* = 0.005).

**Table 1:** Characteristics and clinical outcomes of patients with VV-ECMO or VA-ECMO.

	**VV-ECMO** **(*n* = 15)**	**VA-ECMO** **(*n* = 39)**
Age (year)	64 (51–71)	63 (52–69)
Height (cm)	164 (159–168)	168 (157–174)
Weight (kg)	62.9 (57.0–75.2)	67.4 (56.3–82.3)
Sex (female/male)	6/9	7/32
APACHE II score	31 (26–40)	29 (24–36)
SAVE score	-	−8 (−10 to −2)
RESP score	0 (−3 to 4)	-
RRT, n (%)	4 (27%)	22 (56%)
**ECMO indication**		
E-CPR	-	16
Heart failure	-	17
Postcardiotomy	-	4
Septic shock	-	2
ARDS	15	-
**EAA level**		
T1 median (IQR)	0.44 (0.31–0.71)	0.33 (0.22–0.50)
T2 median (IQR)	0.50 (0.36–0.66)	0.38 (0.28–0.52)
T3 median (IQR)	0.50 (0.33–0.78)	0.40 (0.23–0.57)
T4 median (IQR)	0.49 (0.37–0.59)	0.44 (0.29–0.56)
ECMO free days^a^	0 (0–17)	14 (0–24)
ICU free days^b^	0	0 (0–2)
30-day survival, *n* (%)	11 (73%)	21 (54%)

*Data are presented as medians (interquartile ranges, IQRs), numbers, and percentages as appropriate. Four study time points: within 24 h (T1) and 25–48 (T2), 49–72 (T3), and 73–96 h (T4) after ECMO initiation. APACHE II, Acute Physiology and Chronic Health Evaluation II; ARDS, acute respiratory distress syndrome; EAA, endotoxin activity assay; ECMO, extracorporeal membrane oxygenation; E-CPR, extracorporeal cardiopulmonary resuscitation; ICU, intensive care unit; IQR, interquartile range; RRT, renal replacement therapy; SAVE, Survival after Venoarterial ECMO; RESP, Respiratory ECMO Survival Prediction; VA-ECMO, venoarterial ECMO; VV-ECMO, venovenous ECMO*.

a *ECMO free days were defined as days free from ECMO support, between ECMO initiation and day 30*.

b *ICU free days were defined as days bot in the ICU, between ECMO initiation and day 30*.

### Clinical and Laboratory Data in 30-Day Survivors and Non-survivors

The EAA levels and clinical and laboratory data for VA-ECMO patients surviving or not surviving at 30 days are summarized in Table 2. Significantly higher APACHE II scores and SAVE scores at VA-ECMO initiation were found in 30-day non-survivors. In VA-ECMO patients, EAA levels at T3 were higher in 30-day non-survivors than in survivors (median [interquartile range]: 0.49 [0.37–0.93] vs. 0.31 [0.19–0.51], median difference 0.16 [95% CI, 0.02–0.31]; *P* = 0.024). Biomarker levels are presented in Table 3. Procalcitonin levels at T3 were higher in 30-day non-survivors than in survivors.

**Table 2:** Data of 30-day survivors and non-survivors with VA-ECMO.

**VA-ECMO**	**30-day survivors (n = 21)**	**30-day non-survivors (n = 18)**	***P* values**
Age (year)	65 (55–71)	62 (49–66)	0.367
Sex (female/male)	2/19	5/13	
APACHE II score	27 (24–32)	36 (27–39)	0.020
SAVE score	−5 (−9–−1)	−10 (−15–−7)	0.003
ECMO free days^a^	22 (17–26)	1 (0–13)	0.001
ICU free days^b^	0 (0–15)	0	0.009
**T1 (within 24 h)**			
EAA	0.38 (0.20–0.51)	0.29 (0.20–0.46)	0.319
WBC (k/μL)	12.2 (9.5–17.4)	12.1 (9.7–15.9)	0.988
Inotropic score	4.1 (0–20.7)	12.9 (1.9–19.4)	0.483
Lactate (mmol/L)	10.1 (3.4–13.5)	9.5 (7.7–13.1)	0.478
**T2 (25–48 h)**			
EAA	0.38 (0.20–0.52)	0.38 (0.33–0.52)	0.660
WBC (k/μL)	12.1 (10.6–15.3)	12.8 (8.8–15.6)	0.885
Inotropic score	4.6 (0.7–11.5)	9.2 (3.3–18.8)	0.147
Lactate (mmol/L)	2.9 (1.7–4.5)	3.1 (2.0–4.4)	0.641
**T3 (49–72 h)**			
EAA	0.31 (0.19–0.51)	0.49 (0.37–0.63)	0.024
WBC (k/μL)	12.3 (9.0–15.2)	10.4 (7.9–12.2)	0.128
Inotropic score	2.3 (0.0–8.1)	7.5 (1.1–13.9)	0.220
Lactate (mmol/L)	1.9 (1.4–3.0)	2.5 (1.8–5.3)	0.056
**T4 (73–96 h)**			
EAA	0.41 (0.29–0.52)	0.49 (0.29–0.61)	0.391
WBC (k/μL)	10.1 (8.8–13.5)	11.3 (7.9–11.9)	0.977
Inotropic score	0.9 (0–7.9)	3.2 (0–15.4)	0.427
Lactate (mmol/L)	1.8 (1.2–2.5)	2.5 (1.4–4.0)	0.126

*Data are presented as medians (interquartile ranges), numbers, and percentages as appropriate. Study time points (T1–T4) represent the time after ECMO initiation. APACHE II, Acute Physiology and Chronic Health Evaluation II; EAA, endotoxin activity assay; ECMO, extracorporeal membrane oxygenation; ICU, intensive care unit; SAVE, Survival after Venoarterial ECMO; VA-ECMO, venoarterial ECMO; WBC, white blood cell*.

a *ECMO free days were defined as days free from ECMO support from ECMO initiation until day 30*.

b *ICU free days were defined as days bot in the ICU, between ECMO initiation and day 30*.

**Table 3:** Biomarker levels in 30-day survivors and non-survivors with VA-ECMO.

**VA-ECMO**	**30-day survivors**	**30-day non-survivors**	***P* values**
**T1 (within 24 h)**			
Procalcitonin	4 (1–17)	8 (4–15)	0.442
DAO	14 (8–33)	18 (8–76)	0.419
Cystatin C	0.7 (0.4–1.0)	1.0 (0.3–1.3)	0.338
**T2 (25–48 h)**			
Procalcitonin	4 (1–13)	10 (4–15)	0.165
DAO	9 (5–26)	13 (5–23)	0.705
Cystatin C	0.6 (0.5–1.0)	1.0 (0.4–1.2)	0.338
**T3 (49–72 h)**			
Procalcitonin	3 (1–5)	8 (3–12)	0.022
DAO	5 (4–11)	6 (4–13)	0.752
Cystatin C	0.7 (0.4–1.0)	0.9 (0.5–1.2)	0.209
**T4 (73–96 h)**			
Procalcitonin	2 (0–7)	4 (2–8)	0.202
DAO	6 (4–9)	6 (3–12)	0.667
Cystatin C	0.6 (0.4–1.1)	1.0 (0.6–1.1)	0.131

*Data are presented as medians (interquartile ranges), numbers, and percentages as appropriate. Study time points (T1–T4) represent the time after ECMO initiation. DAO, diamine oxidase; VA-ECMO, venoarterial ECMO*.

### *Post hoc* Analysis: Clinical and Laboratory Data in VA-ECMO Patients With or Without Elevated EAA Levels at T3

**Figure 3:**
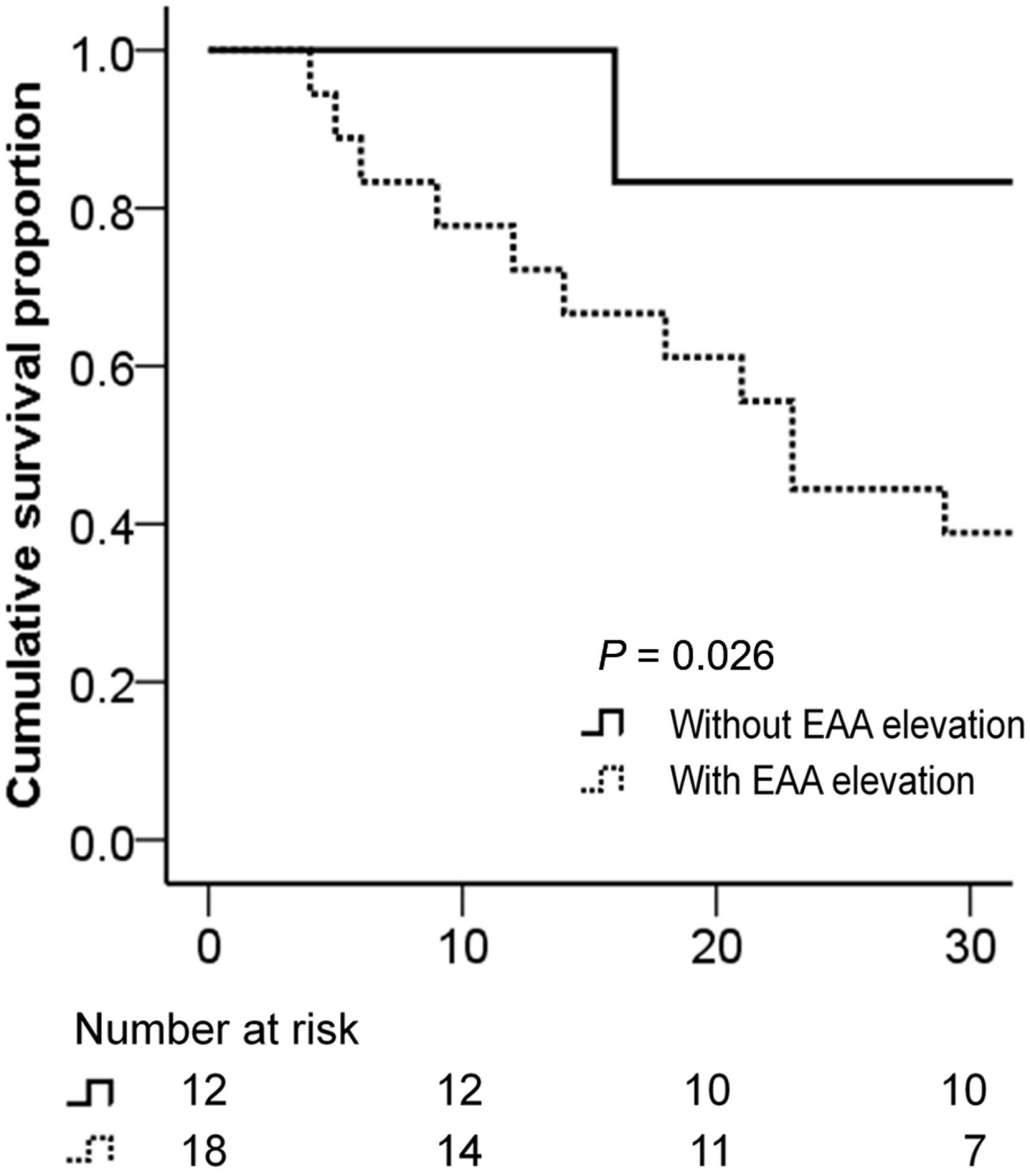
Kaplan–Meier 30-day survival curve for VA-ECMO patients. Patients with or without elevated EAA level at T3 (vs. T1). Study time points: within 24 h (T1) and 49–72 (T3) after ECMO initiation. Result of statistical comparison using log-rank test. EAA, endotoxin activity assay; VA-ECMO, venoarterial extracorporeal membrane oxygenation.

The EAA levels and clinical and laboratory data of VA-ECMO patients with or without elevated EAA levels at T3 (vs. T1) are summarized in Table 4. In VA-ECMO patients, patients with an elevated EAA level at T3 (vs. T1) had lower 30-day survival (39 vs. 83%, *P* = 0.026) and fewer ECMO free days by day 30 (median [interquartile range]: 3 [0–21] vs. 23 [15–25] days, median difference 12 days [95% CI, 0–22]; *P* = 0.028) than did those without an elevated EAA level. Biomarker levels are presented in Table 5. The DAO levels at T1 and the WBC counts at T4 were higher in VA-ECMO patients with an elevated EAA level than in those without. The 30-day survival curves for VA-ECMO patients with or without elevated EAA levels at T3 are presented in Figure 3. The EAA levels in VA-ECMO patients with a proven infection within 96 h are presented in Table 6.

**Table 4:** Data of VA-ECMO patients with or without elevated EAA levels at T3.

**VA-ECMO**	**Δ EAA _**T3−T1**_ < 0 (*n* = 12)**	**Δ EAA _**T3−T1**_ ≥ 0(*n* = 18)**	***P*-values**
Age (year)	66 (58–71)	63 (48–68)	0.439
SAVE score	−5 (−10 to −1)	−10 (−11 to −4)	0.232
APACHE II score	30 (25–37)	28 (24–36)	0.692
ECMO indication			
E-CPR	4	6	
Heart failure	7	8	
Postcardiotomy	0	3	
Septic shock	1	1	
ECMO free days^a^	23 (15–25)	3 (0–21)	0.028
ICU free days^b^	0 (0–3)	0 (0–4)	0.602
30-day survival	10 (83%)	7 (39%)	0.026
T1 Number, *n* (%)	12 (40%)	18 (60%)	
EAA	0.42 (0.24–0.51)	0.28 (0.17–0.42)	0.146
WBC (k/μL)	12.9 (10.3–15.2)	11.9 (9.1–15.9)	0.602
Inotropic score	9.9 (3.9–30.6)	3.7 (0–15.8)	0.215
Lactate (mmol/L)	9.0 (3.4–13.2)	9.2 (7.0–12.5)	0.662
T2 Number, *n* (%)	12 (41%)	17 (59%)	
EAA	0.25 (0.12–0.47)	0.46 (0.35–0.52)	0.034
WBC (k/μL)	11.3 (9.3–13.0)	12.9 (11.3–16.9)	0.087
Inotropic score	4.8 (2.4–9.1)	7.6 (0.0–22.1)	0.647
Lactate (mmol/L)	2.5 (1.7–2.9)	3.3 (1.5–5.0)	0.245
T3 Number, *n* (%)	12 (40%)	18 (60%)	
EAA	0.20 (0.11–0.30)	0.51 (0.38–0.60)	0.001
WBC (k/μL)	9.0 (7.5–12.5)	11.4 (8.9–13.2)	0.146
Inotropic score	4.9 (1.7–8.5)	5.4 (0.0–15.9)	0.723
Lactate (mmol/L)	1.8 (1.4–2.5)	2.9 (1.4–5.0)	0.180
T4 Number, *n* (%)	11 (41%)	16 (59%)	
EAA	0.32 (0.21–0.51)	0.48 (0.30–0.52)	0.195
WBC (k/μL)	8.8 (6.8–10.5)	11.5 (9.4–12.8)	0.019
Inotropic score	1.6 (0–6.7)	1.6 (0.0–13.1)	1.000
Lactate (mmol/L)	1.8 (1.2–3.1)	2.1 (1.1–3.2)	0.645

*Data are presented as medians (interquartile ranges), numbers, and percentages as appropriate. Four study time points: within 24 h (T1) and 25–48 (T2), 49–72 (T3), and 73–96 h (T4) after ECMO initiation. APACHE II, Acute Physiology and Chronic Health Evaluation II; EAA, Endotoxin Activity Assay; ECMO, extracorporeal membrane oxygenation; ICU, intensive care unit; SAVE, Survival after Venoarterial ECMO; VA-ECMO, venoarterial ECMO; WBC, white blood cell*.

a *ECMO free days were defined as days free from ECMO support from ECMO initiation until day 30*.

b *ICU free days were defined as days bot in the ICU, between ECMO initiation and day 30*.

**Table 5:** Biomarker levels in VA-ECMO patients with or without elevated EAA levels at T3.

**VA-ECMO**	**Δ EAA _**T3-T1**_ < 0**	**Δ EAA _**T3-T1**_ ≥ 0**	***P* values**
**T1 (within 24 h)**			
Procalcitonin	3 (1–16)	7 (3–16)	0.632
DAO	8 (7–21)	28 (13–76)	0.013*
Cystatin C	1 (0.6–1.2)	0.6 (0.3–1.0)	0.079
**T2 (25–48 h)**			
Procalcitonin	5 (2-13)	8 (2–19)	0.444
DAO	5 (4–17)	14 (6–23)	0.117
Cystatin C	1 (0.6–1.2)	0.5 (0.3–1.0)	0.080
**T3 (49–72 h)**			
Procalcitonin	2 (1–5)	8 (3–12)	0.065
DAO	5 (3–20)	6 (4–9)	0.851
Cystatin C	0.9 (0.5–1.3)	0.8 (0.4–1.0)	0.124
**T4 (73–96 h)**			
Procalcitonin	2 (1–4)	5 (2–10)	0.241
DAO	5 (4–14)	5 (4–8)	0.698
Cystatin C	0.9 (0.5–1.2)	0.7 (0.4–1.1)	0.100

Data are presented as medians (interquartile ranges), numbers, and percentages as appropriate. DAO; diamine oxidase; EAA, endotoxin activity assay; VA-ECMO, veno-arterial ECMO. *significant difference between patients with or without elevated EAA level from T1–T3. Study time points (T1–T4) represent the time after ECMO initiation.

**Table 6:** EAA levels in VA-ECMO patients with a proven infection within 96 h.

**No**.	**Type**	**Microorganisms**	**Day**	**EAA**			
				**T1**	**T2**	**T3**	**T4**
4	Bacteremia	*Actinomyces odontolyticus*	0	0.38	0.35	0.59	0.49
10	Pneumonia	*Klebsiella pneumoniae*	1	0.47	0.57	0.47	0.52
14	Bacteremia	*Klebsiella pneumoniae*	0	-	0.57	0.76	0.61
26	Pneumonia	*Klebsiella pneumoniae*	0	0.17	0.38	0.52	0.56
31	Pneumonia	*Stenotrophomonas maltophilia*	3	0.49	0.35	0.61	0.5
37	Pneumonia	*Klebsiella pneumoniae*	0	0.26	0.52	0.9	0.59
44	Pneumonia	*Acinetobacter nosocomialis*	4	0.17	0.14	0.38	0.29
45	Pneumonia	*Klebsiella pneumoniae*	4	0.13	0.81	0.24	0.32
46	Pneumonia	*Klebsiella pneumoniae*	0	0.5	0.11	0.05	-
47	Pneumonia	*Elizabethkingia meningoseptica*	3	0.21	0.39	0.16	0.29
48	Pneumonia	*Pseudomonas aeruginosa*	4	0.4	0.35	0.4	-
51	Pneumonia	*Klebsiella pneumoniae*	0	-	0.36	0.28	0.32

*Four study time points: within 24 h (T1) and 25–48 (T2), 49–72 (T3), and 73–96 h (T4) after ECMO initiation. EAA, endotoxin activity assay*.

## Discussion

This study discovered that 42% of VV-ECMO patients and 9% of VA-ECMO patients had high EAA levels (≥0.6) at the initiation of ECMO support. In VA-ECMO patients, EAA levels at T3 were higher in 30-day non-survivors than in survivors. VA-ECMO patients with elevated EAA levels at T3 (vs. T1) had lower 30-day survival than those without elevated EAA levels had.

Our study demonstrated that incidence of high EAA levels (≥0.6) was higher in VV-ECMO patients than in VA-ECOM patients; this finding is compatible with a report that patients with VV-ECMO have a higher risk of infection than do those with VA-ECMO (13). It has been reported that 63% of ventilator-associated pneumonia were due to gram-negative bacteria (GNB) (14), and the GNB sepsis directly result in high endotoxin activity. However, in patients with non-GNB sepsis, severe hypoperfusion or hypoxemia could damage the intestinal barrier and results in the translocation of intestinal resident bacteria (mainly GNB) and endotoxins (5). Therefore, the translocation could secondarily result in high endotoxin toxicity in these patients with non-GNB sepsis. Moreover, current surviving sepsis guidelines suggest using VV-ECMO for patients with sepsis-induced severe ARDS when conventional mechanical ventilation fails (15). We suggest that more concerns should be raised for high endotoxin activity in VV-ECMO patients, and further investigations of the impact of high endotoxin activity on clinical outcomes are needed.

Elevated EAA levels at T3 were associated with lower 30-day survival and fewer ECMO-free days in VA-ECMO patients of this study. Several factors may contribute to subsequent elevated endotoxin activity in the whole blood of VA-ECMO patients. First, ICU-related nosocomial infections (e.g., ventilator-associated pneumonia and bloodstream infection) are common prior to and during ECMO support (14, 16, 17). Among the 39 VA-ECMO patients in the present study, six presented with infections prior to ECMO initiation, and nine developed infections within the first week after ECMO initiation. Most microorganisms reported within 96 h after VA-ECMO placement in this study were GNB. Second, microcirculatory dysfunction may disrupt the intestinal barrier and precipitate the translocation of resident intestinal bacteria and endotoxins (5, 18). Third, we found that DAO levels at T1 were higher in VA-ECMO patients with an elevated EAA level at T3 than in those without. This finding might be associated with intestinal barrier disruption. Moreover, several reports suggest that some *Klebsiella pneumoniae* infections originate from the gastrointestinal reservoir (19, 20), which is compatible with the finding of this study that *Klebsiella pneumoniae* is the most common pathogen. Furthermore, extracorporeal endotoxin adsorption treatment has been reported to reduce EAA and inflammatory cytokine levels (21, 22). Further studies are warranted to investigate the clinical efficacy of extracorporeal endotoxin adsorption treatment in VA-ECMO patients with elevated EAA levels.

This study has several limitations. First, with limited sample size, the results of exploratory analyses require further investigation in future studies. Second, the mechanism of increased endotoxin activity may vary among patients with different indications for VA-ECMO support. Further studies are warranted to investigate such variation. Third, an aggravated systemic inflammatory response resulting from the exposure of blood to extracorporeal circulation has been recognized as an independent cause of intestinal barrier dysfunction in porcine models (23, 24). Further studies are warranted to investigate the relationship between cytokine level and endotoxin activity.

## Conclusions

A certain proportion of critically ill adult patients with ECMO have an EAA level ≥0.6, and an elevated EAA levels at 48 h after VA-ECMO initiation was associated with lower 30-day survival. Further studies are warranted to investigate the risk factors for high endotoxin activity and the effects of high endotoxin activity on further clinical outcomes in larger patient groups. Additionally, new therapeutic strategies for high endotoxin activity are required to be investigated.

## Data Availability Statement

The raw data supporting the conclusions of this article will be made available by the authors, without undue reservation.

## Ethics Statement

The studies involving human participants were reviewed and approved by Research Ethics Committee of National Taiwan University Hospital. The legally authorized representatives of the patients provided their written informed consent to participate in this study.

## Author Contributions

C-TL, C-HW, W-SC, and Y-CY: concept and design and drafting manuscript. C-HW, C-HL, and Y-CY: patient enrollment and data collection. T-JW, W-SC, M-JW, and Y-CY: interpretation of data. C-HW, M-JW, Y-SC, and Y-CY: critical revision of the manuscript. M-JW and Y-SC: study supervision. All authors contributed to the article and approved the submitted version.

## Funding

This work was supported, in part, by a grant from the National Taiwan University Hospital (NTUH 109-S4468).

## Conflict of Interest

The authors declare that the research was conducted in the absence of any commercial or financial relationships that could be construed as a potential conflict of interest.

## Publisher's Note

All claims expressed in this article are solely those of the authors and do not necessarily represent those of their affiliated organizations, or those of the publisher, the editors and the reviewers. Any product that may be evaluated in this article, or claim that may be made by its manufacturer, is not guaranteed or endorsed by the publisher.
